# Supplementary material for: The role of family conflict and cohesion in adolescents’ social responsibility: Emotion regulation ability as a mediator
Source: PLoS One. 2024 Sep 30;19(9):e0311265. doi: 10.1371/journal.pone.0311265 (PMC11441698; doi:10.1371/journal.pone.0311265)
Supplement: S1 Appendix — (DOCX) [file pone.0311265.s002.docx]

TITLE: family-ER-social responsibility;

DATA:

File is C:\Users\cheun\Documents\Macau\Sandy\data.csv;

VARIABLE: NAMES ARE

ei_roe_c !this variable denotes emotion regulation

fes_co_c !this variable denotes family cohesion

fes_cf_c !this variable denotes family conflict

srm !this variable denotes social responsibility

sex !this variable denotes gender

age !this variable denotes age

income !this variable denotes family income

sib !this variable denotes number of siblings

stm !this variable denotes social trust

;

Missing are all(999);

Usevariables are

ei_roe_c

fes_co_c

fes_cf_c

!coh_roe

!cof_roe

srm

sex

age

income

sib

stm;

ANALYSIS:

BOOTSTRAP 10000;

MODEL:

ei_roe_c ON

fes_co_c

fes_cf_c

sex

age

income

sib;

srm ON

ei_roe_c

fes_co_c

fes_cf_c

stm

sex

age

income

sib;

sex with age@0;

sex with income@0;

sex with sib@0;

STM WITH sex@0;

STM with income@0;

stm with sib@0;

stm with age@0;

age with income@0;

age with sib@0;

income with sib@0;

FES_CO_C WITH STM ;

FES_CF_C WITH FES_CO_C;

INCOME WITH EI_ROE_C;

model indirect:

srm IND fes_co_c;

srm IND fes_cf_c;

OUTPUT: sampstat stand tech4 modindices STDYX;

CINTERVAL (BCBOOTSTRAP);
