# Supplementary material for: The role of family conflict and cohesion in adolescents’ social responsibility: Emotion regulation ability as a mediator
Source: PLoS One. 2024 Sep 30;19(9):e0311265. doi: 10.1371/journal.pone.0311265 (PMC11441698; doi:10.1371/journal.pone.0311265)
Supplement: Attachment — Submitted filename: Point-by-point responses_2024-8-29_FINAL.docx [file pone.0311265.s003.docx]

**Family Processes and Adolescents’ Social Responsibility: The Mediating Role of Emotion Regulation**

Editor’s comments:

- *The methodology and analysis are brief, but the findings and Chinese data may have important implications for the research context of family psychology.*
  - Thank you for your comment. We have now provided further details in the methodology and analysis to enrich the manuscript.
- *As the reviewer pointed out, the sections about data analysis and discussion should be improved.*
  - We have revised the data analysis section and the discussion according to the reviewer’s recommendation. Please see our responses below for the details. Thank you.
- *Hypotheses should be written clearly.*
  - Thank you for your comment. We have revised the hypotheses in the manuscript as follows, “We hypothesized that family cohesion and family conflict would have a direct effect on social responsibility, and the effects would be mediated by emotional regulation ability. Specifically, we hypothesized that family cohesion would be positively associated with emotion regulation ability, and family conflict would be negatively associated with emotion regulation ability. Emotion regulation ability, in turn, would be positively associated with social responsibility. Taken together, we hypothesized that emotion regulation ability would mediate the effect of family cohesion and family conflicts on social responsibility.” (lines 131-138)
- *The data analysis section is too short. Although the data file is available, the analysis code should also be available to improve reproducibility. A more detailed supplementary explanation for how to read the data file (e.g., "999" means a missing value) would also be better.*
  - Thank you for the comment. We have revised the data analysis section (lines 214-216), “Prior to data analyses, Little’s Missing Completely at Random (MCAR) test was conducted via SPSS to examine the pattern of missing values and determine how the missingness should be treated.” In the results section, we have added (lines 236-238), “Regarding the missing data analysis, the MCAR test was not significant, χ^2^(14) = 22.16, *p* = .075, indicating that the data were completely missing at random. As such, full information maximum likelihood was used to handle the missing data.”

To enable the readers to understand the analysis and read the data file, we have added the following in the manuscript (lines 216-222), “Path analysis was conducted via MPLUS, Version 8.3 [58] to investigate the emotion regulation ability as a mediator for the effects of family cohesion and family conflict on adolescents’ social responsibility, over and above the covariates identified in previous research (i.e., age, gender, family income, number of siblings, and social trust). Mediation was tested by using 10,000 bootstrapping iterations to obtain the bootstrapped confidence intervals of the indirect effects. The data file in the Appendix includes the variables under study, with a score of “999” denotes a missing value. The MPLUS codes are included in the appendix.”

- *You should discuss the results regarding the reason for the positive relationship between family conflict and social responsibility, although this may be irrelevant to the hypothesis.*
  - Thank you for your comment. We added the discussion on this finding, “We found that higher levels of family cohesion and family conflict were both significantly associated with adolescents’ higher level of social responsibility. The finding on the positive relationship between family cohesion and adolescents’ social responsibility was consistent with previous studies [18, 21]. As for the surprising positive association between family conflict and social responsibility, we speculate that adolescents with higher family conflict might have sought recognition outside their families, such as from peers and social groups, which could motivate them to develop a stronger sense of social responsibility [59]. As such, future studies should include third variables, such as peer and social support, to understand the relation between family conflict and social responsibility.” (lines 299-307)

Journal requirements:

- *Please ensure that your manuscript meets PLOS ONE's style requirements, including those for file naming. The PLOS ONE style templates can be found at* 
  [*https://journals.plos.org/plosone/s/file?id=wjVg/PLOSOne_formatting_sample_main_body.pdf*](https://journals.plos.org/plosone/s/file?id=wjVg/PLOSOne_formatting_sample_main_body.pdf) *and* 
  [*https://journals.plos.org/plosone/s/file?id=ba62/PLOSOne_formatting_sample_title_authors_affiliations.pdf*](https://journals.plos.org/plosone/s/file?id=ba62/PLOSOne_formatting_sample_title_authors_affiliations.pdf)
- Thank you for the reminders. We have reformatted the manuscript to meet the requirements.
- *Thank you for stating the following financial disclosure: 
  “The present study was funded by The Education University of Hong Kong and the Centre for Child and Family Science at The Education University of Hong Kong (R3669 & CCFS2017-0003).” Please state what role the funders took in the study.  If the funders had no role, please state: “The funders had no role in study design, data collection and analysis, decision to publish, or preparation of the manuscript.”*
  - Thank you for the reminders. In the cover letter, we have now stated, “The funders had no role in study design, data collection and analysis, decision to publish, or preparation of the manuscript.”
- *Please include your full ethics statement in the ‘Methods’ section of your manuscript file. In your statement, please include the full name of the IRB or ethics committee who approved or waived your study, as well as whether or not you obtained informed written or verbal consent. If consent was waived for your study, please include this information in your statement as well.*
- The full ethics statement has been included in the ‘Methods’ section of the manuscript file, “The study was conducted with the approval of the Ethics Committee of The Education University of Hong Kong. Written informed consent and assent were obtained from the adolescents and their parents prior to their participation in the study.” (lines 153-155).
- *Please review your reference list to ensure that it is complete and correct. If you have cited papers that have been retracted, please include the rationale for doing so in the manuscript text, or remove these references and replace them with relevant current references. Any changes to the reference list should be mentioned in the rebuttal letter that accompanies your revised manuscript. If you need to cite a retracted article, indicate the article’s retracted status in the References list and also include a citation and full reference for the retraction notice.*
- Thank you for your reminder. We have reviewed the reference list.

Reviewer’s comments:

- *The manuscript is written in a clear and coherent way and is based on data collected from a large sample.*
  - Thank you for the positive comment.
- *The title is misleading. It is hard to understand what “family process” refers to.*
  - Thank you for your comment. We revised the title to, “The Role of Family Conflict and Cohesion in Adolescents’ Social Responsibility: Emotion Regulation Ability as a Mediator”
- *The abstract is not informative enough. The authors have tested a mediation model. It is hard to understand this by reading the abstract.*
  - Thank you for the comment. The abstract has been revised to emphasize we have tested a mediation model. It reads (lines 3-5), “To understand the role of social context in adolescent development, the present study examined family predictors (i.e., family cohesion and conflict) of social responsibility, with emotion regulation ability as a mediating process.”
- *Introduction - The following sentence can be revised “As another index of the family environment, family conflict indicates the extent of anger, aggression, and conflictual interactions between family members”.*
  - Thank you for the comment. We deleted “As another index of the family environment” to avoid confusion. It now reads (lines 67-68), “Family conflict indicates the extent of anger, aggression, and conflictual interactions between family members [25].”
- *The authors state that the study is based on family systems theory and the ecological framework. However, they paid little attention to these models. The introduction should be reorganized by putting more emphasis on its theoretical basis.*
  - Thank you for your comment. We have now elaborated on the theoretical frameworks in the Introduction: “Together with the socioecological framework [15] and family systems theory [16], family is postulated to be an important environment for adolescent development, particularly in a Chinese context. According to Bronfenbrenner’s socioecological framework [15], parental practices have a direct influence on child and adolescent socioemotional development. As an active agent, children also affect their social environment, creating dynamics in the social ecologies. Similarly, family systems theory [16] describes the family as an organized system composed of interdependent subsystems such as the interparental subsystem and the parent-child subsystem [6, 15]. The theory highlights the dynamic interaction between family members, generating mutual influences within the family that foster adolescent development [17]. Based on these theoretical frameworks, in this study we propose that the interplay between family relationships and adolescents could facilitate adolescent development of emotion regulation ability and social responsibility [6, 15]. ” (lines 45-56)
- *The concept “social responsibility” needs to be clearly explained.*
  - We have now defined the concept of social responsibility in the first paragraph, “Social responsibility is a value orientation that reflects people’s concern for others extending beyond personal interest [3]. Social responsibility also reflects people’s sense of duty to address social needs [4]. For instance, an individual who has a strong sense of social responsibility might have a stronger value for helping those who are less fortunate, working hard to improve society, donating time or money to charities, and engaging in social movements that benefit the country [5].” (lines 22-27)
- *The following sentence is difficult to follow “The relationship between family conflict and emotion regulation was also examined with impulsivity (34), and the findings suggested that ongoing family conflict was related to an increased risk for greater emotional negativity and greater aggression over time (34, 35)”.*
  - Thank you for your comment. We have deleted the sentence, “The relationship between family conflict and emotion regulation was also examined with impulsivity (34), and the findings suggested that ongoing family conflict was related to an increased risk for greater emotional negativity and greater aggression over time (34, 35)”. to avoid confusion, as they were not directly related to our research question.
- *The section on the mediator role of ER can be reorganized by making sure that the authors cover only the literature directly related to the current study. For example, the have cited studies that focus on empathy which is indirectly related to the current research question. Also, the study that I have mentioned above on impulsivity seems indirectly related.*
  - Thank you for the suggestion. We have trimmed down the studies that were not directly related to the research question to avoid confusion, including studies revolving around empathy and impulsivity.
- *Although the authors have provided a definition of ER. However, an examination of previous studies indicated that there are multiple ways of examining ER. Certain studies approach ER based on the use of specific strategies. Some others focus on difficulties in ER or ER flexibility. It is thus, important to explain the specific ER framework utilized in the present study.*
  - Thank you for your comment. We clarified the definition of ER in the present study, “There are multiple ways of examining emotion regulation. Previous studies have adopted the process model of emotional regulation [34] and examined emotion regulation strategies, such as attentional deployment, cognitive change, and response modulation. Nevertheless, other studies adopted a different approach and emphasized emotion regulation ability [35, 36], i.e., an individual’s ability to regulate their emotions for recovering quickly from psychological distress [37]. Given that emotion regulation ability is associated with adaptation in social relationships and academic performance [38-40], this study examined the role of an overall emotion regulation ability, rather than specific emotion regulation strategies.” (lines 96-103)
- *Family process, family cohesion, family precursor are different names used with reference to the same concept. It may be a good idea to be consistent while using terms.*
  - Thank you for your suggestion. We have now minimized the use of multiple terms to increase consistency.
- *At the end of the introduction the authors stated, “Social trust was also examined as the covariate of social responsibility”. This concept appears for the first time in the final sentence of the introduction section. The authors have provided a justification. However, the justification is not enough to explain why the authors have included social trust as a covariate. More explanation is needed.*
  - Thank you for your suggestion. We have elaborated on social trust in the Introduction, “Adolescents’ social trust might further obscure the link between family factors and social responsibility. Social trust is defined as an individual’s belief in the fairness and trustworthiness of their treatment within the society [30]. Previous research suggested that family cohesion was positively related to higher social trust among Chinese adolescents [31]. Another study found that family cohesion was related to social responsibility over and above the effect of social trust [18], suggesting that social trust may be linked to family cohesion and social responsibility. Therefore, to identify the unique correlates of social responsibility, we examined the links between family conflict, cohesion, and social responsibility, over and above social trust as covariate.” (lines 83-91)
- *The hypotheses should be written more clearly, I did not understand that both family cohesion and family conflict act as the independent variables until I started reading the results section.*
  - Thank you for your comment. The hypotheses have been rewritten, “We hypothesized that family cohesion and family conflict would have a direct effect on social responsibility, and the effects would be mediated by emotional regulation ability. Specifically, we hypothesized that family cohesion would be positively associated with emotion regulation ability, and family conflict would be negatively associated with emotion regulation ability. Emotion regulation ability, in turn, would be positively associated with social responsibility. Taken together, we hypothesized that emotion regulation ability would mediate the effect of family cohesion and family conflicts on social responsibility.” (lines 131-138)
- *The final paragraph of introduction should give certain cues regarding the potential contribution of the present study to the literature.*
  - Thank you for your comment. We have added the potential contribution of the present study, “Extending beyond the current theoretical understanding of adolescent development, potential findings could provide initial evidence of emotion regulation as a process for the effects of family cohesion and family conflict on adolescents’ social responsibility. Beyond theoretical contributions, the findings could also inform the design and implementation of interventions aimed at promoting adolescents’ social responsibility.” (lines 141-146)
- *Method - The participants section is well-written and informative. However, I have concerns regarding the presentation of t-tests and chi-square results in the participants section rather than the results section.*
  - Thank you for the comment. We have moved the presentation of t-tests and chi-square results to the Results section (lines 226-235).
- *The authors, I assume, used the Chinese versions of the scales mentioned. It is crucial to report the psychometric properties of the Chinese version of the scales. Are they culturally appropriate, were there any problems in the adaptation process etc.
  It may be a good idea to use the term “emotion regulation ability” rather than just “emotion regulation”.*
  - Thank you for your comment. We used the Chinese versions of the scales. We have mentioned the psychometric properties of the measures under study (lines 173-212). Given that the Social Responsibility Scale [5] and the Social Trust Scale [57] had not undergone a validation or adaptation process in Chinese samples, as a limitation, we have now stated, “Given the poor internal consistency of the Social Trust Measure [57], validation studies should be conducted to ensure the measure is valid for use in Chinese samples. Cautions should also be made in interpreting the present findings and replication studies should be conducted in the future. Similarly, the Social Responsibility Scale has not been validated in the Chinese context. Future research should conduct validation studies to assess its factor structure and validity.” (lines 358-363)
  - We have now updated the term “emotion regulation” to “emotional regulation ability” throughout the manuscript
- *The authors have used quite brief versions of the scales. I wonder whether they are efficiently assessing the constructs that form the study variables.*
  - Thank you for the comment. We acknowledge that the measures under study were brief. However, the Family Environment Scale and the Emotional Intelligence Scale had demonstrated adequate internal consistency, factor structure, and intercorrelations between subscales in both Chinese and Western samples in previous studies [52, 53, 54]. Even though the measures of social responsibility had demonstrated adequate internal consistency in previous Chinese samples [18], we recognize that the measure had not been validated in the Chinese context. As such, we have included a limitation, “Given the poor internal consistency of the Social Trust Measure [57], validation studies should be conducted to ensure the measure is valid for use in Chinese samples. Cautions should also be made in interpreting the present findings and replication studies should be conducted in the future. Similarly, the Social Responsibility Scale has not been validated in the Chinese context. Future research should conduct validation studies to assess its factor structure and validity.” (lines 362-363)
- *Data analysis section should be more detailed.*
  - Thank you for the comment. We have revised the data analysis section: “Prior to data analyses, Little’s Missing Completely at Random (MCAR) test was conducted via SPSS to examine the pattern of missing values and determine how the missingness should be treated. Path analysis was conducted via MPLUS, Version 8.3 [58] to investigate the emotion regulation ability as a mediator for the effects of family cohesion and family conflict on adolescents’ social responsibility, over and above the covariates identified in previous research (i.e., age, gender, family income, number of siblings, and social trust). Mediation was tested by using 10,000 bootstrapping iterations to obtain the bootstrapped confidence intervals of the indirect effects. The data file in the Appendix includes the variables under study, with a score of “999” denotes a missing value. The MPLUS codes are included in the appendix. Given the low internal consistency of the three-item Social Trust measure, supplementary path analysis was conducted using a two-item social trust measure that demonstrated a higher internal consistency.” (lines 214-224)
- *Results - Correlation results are provided via the Table 2, but there is no section in the results section explain the results of correlation analyses.*
- Thank you for your comment. We added the results of correlation analyses in the Results section. “The zero-order correlations among all variables are presented in Table 2. Family cohesion was negatively correlated with family conflict (*r* = -.58, *p* < .001) and positively correlated with adolescents’ emotion regulation ability (*r* = .20, *p* < .001), social responsibility (*r* = .22, *p* < .001), and social trust (*r* = .23, *p* < .001). Family conflict was negatively correlated with adolescents’ emotion regulation ability (*r* = -.24, *p* < .001), social responsibility (*r* = -.08, *p* = .02), and social trust (*r* = -.19, *p* < .001). Adolescents’ emotion regulation ability was positively correlated with social responsibility (*r* = .17, *p* < .001) and social trust (*r* = .20, *p* < .001). As for covariates, adolescents’ social responsibility was positively correlated with social trust (*r* = .23, *p* < .001). Adolescents’ age was negatively correlated with family cohesion (*r* = -.14, *p* < .001) and social trust (*r* = -.07, *p* = .04) and positively correlated with family conflict (*r* = .12, *p* < .001). Household income was positively correlated with family cohesion (*r* = .19, *p* < .001) and negatively correlated with family conflict (*r* = -.15, *p* = .002).” (lines 238-250)
- *The following paragraph can be written in a better way “The indirect effects of family cohesion and family conflict to social responsibility via emotion regulation were significant (β = .03, p < .01). Based on 10000 bootstrap samples with replacement, the 95% confidence interval (CI) indicated that the standardized indirect effect from family cohesion and family conflict to adolescents’ social responsibility via emotion regulation did not include a zero [CI: (.03, .19) for family cohesion and (-.14, -.01) for family conflict], thereby suggesting emotion regulation as a mediator.” Coefficients should be added.*
  - Thank you for your comment. We have revised the paragraph by adding the coefficients (lines 274-280).
- *Discussion - There is some repetition in the discussion section. The first two paragraphs highlight the same issues. The authors should write more concisely.*
  - Thank you for your comment. We have revised the first two paragraphs of the Discussion. (lines 287-307)
- *The mediation results should be discussed more thoroughly.*
  - Thank you for your comment. We have elaborated on the findings in this revision.

Regarding the links between family cohesion, family conflict, and adolescents’ emotion regulation, we have stated, “Specifically, our results showed that family cohesion was associated with adolescents’ better emotion regulation ability, whereas family conflict was associated with poorer emotion regulation ability. Consistent with the socioecological framework [15], which states that development occurs as a result of regular exchanges between an individual and the context [15], the findings suggested that the family context is crucial in fostering adolescents’ emotion regulation ability.” (lines 311-316). As for the link between emotion regulation and social responsibility, we have now included, “Our study further indicated that adolescents’ emotion regulation ability was associated with greater social responsibility, which is consistent with previous research [48, 49]. As such, the finding underscores the importance of adolescents’ ability to manage and understand emotions in fostering a sense of social responsibility, such as prioritizing their sense of duty towards group or social needs over personal interest.” (lines 320-325). “ The present findings have implications for fostering adolescents’ social responsibility. Importantly, they suggest that a family environment characterized by higher family cohesion and lower family conflict may enhance adolescents’ emotion regulation ability, which in turn promotes social responsibility. These findings have implications for applied research to involve caregivers, educators, policy makers, and other practitioners in cultivating adolescents’ emotion regulation ability and social responsibility.” (lines 341-346)

- *A Cronbach’s alpha coefficient of .47 is really low. It is a significant limitation that needs to be addressed more clearly. The authors can check the items and look for the item that is responsible for the low internal consistency. In line with one of my previous comments, it can be useful to check the reliability coefficients of the original version and the first Chinese adaptation of the scale as well.*
  - Thank you for your comment. We have conducted supplementary analysis and included these details in the Data Analysis section, “Given the low internal consistency of the three-item Social Trust measure, supplementary path analysis was conducted using a two-item social trust measure that demonstrated a higher internal consistency.” (lines 222-224) In the Results section, we have added, “Supplementary analysis was conducted with a two-item measure of social trust that exhibited a greater internal consistency. However, the mediation model replaced by the two-item measure of social trust did not converge, suggesting potential issues with the computations. As such, predictions were not made in the supplementary analysis. ” (lines 282-285)
  - Given the situation, we have revised the Limitation and Future Directions section, “Third, the Social Trust Measure [57] had a low Cronbach’s alpha at .47, which was a major limitation of this study. Pallant [61] mentioned that measures with fewer than ten items could have low Cronbach’s alphas, which might be the reason behind the low alpha of social trust in our sample. Upon deletion of the item contributing to the low alpha, we conducted additional analysis for this study. However, predictions could not be made as the path model did not converge. Given the poor internal consistency of the Social Trust Measure [57], validation studies should be conducted to ensure the measure is valid for use in Chinese samples. Cautions should also be made in interpreting the present findings and replication studies should be conducted in the future. Similarly, the Social Responsibility Scale has not been validated in the Chinese context. Future research should conduct validation studies to assess its factor structure and validity. ” (lines 353-263)
- *Have the authors checked the participant authenticity?*
  - Thank you for the question. Unfortunately, we did not check the participants’ authenticity. As a limitation, we have now stated, “Second, the variables under study were self-reported, thereby introducing self-report bias revolving around social desirability and a potential lack of authenticity in the reports. Future studies should therefore use a multi-method, multi-informant approach to minimize the potential biases introduced in the study.” (lines 350-353)
- *The data were collected from Chinese adolescents. It would be interesting to read something about the possible impact of culture at least in the discussion section, keeping in mind that this is not a cross-cultural study.*
  - We have added introduction and discussion on the possible impact of culture. In the Introduction, we have added, “In Chinese culture, individuals highly value family relationships and perceive that the family unit is one of the most basic and important social units [12]. Under the influence of Confucianism in the East Asian context, Chinese families emphasize common good and harmony over personal interests [12], and prefer to avoid conflict and maintain harmonious interparental and parent-child relationships [13, 14].” (lines 41-45) In the Discussion, we have added, “In addition, the study was conducted in a Chinese cultural context that emphasizes the importance of family harmony and cohesion as a foundation of the society [12]. We speculated that this cultural context, which values a high-cohesion and low-conflict family environment, might have particularly contributed to cultivating adolescents’ emotion regulation ability and social responsibility. To examine the strength of associations among the variables across cultures, cross-cultural studies between Eastern and Western contexts are necessary in the future.” (lines 334-340)
  - We have also addressed the limitation of the current study now: “Lastly, although the current study discussed the role of Chinese culture on family dynamics, we did not conduct a cross-cultural study or include cultural values as variables under study. Researchers should further examine the role of cultural values in family dynamics and adolescent development, particularly across cultural contexts.” (lines 369-373)
